# Supplementary material for: HIVprotI: an integrated web based platform for prediction and design of HIV proteins inhibitors
Source: J Cheminform. 2018 Mar 9;10:12. doi: 10.1186/s13321-018-0266-y (PMC5845081; doi:10.1186/s13321-018-0266-y)
Supplement: Supplementary file 2 — Additional file 2. Source code of HIVProtI web server. [file 13321_2018_266_MOESM2_ESM.zip › HIVprotI_Source-code/hivprotI/weka-3-6-6/doc/weka/gui/streams/InstanceEvent.html]

InstanceEvent


---


|  |  |  |
| --- | --- | --- |
| **Overview** \| **Package** \| **Class** \| **Tree** \| **Deprecated** \| **Index** \| **Help** \| **Weka's home** | |  |
| **PREV CLASS**   **NEXT CLASS** | **FRAMES**    **NO FRAMES**     **All Classes** |  |
| SUMMARY: NESTED \| FIELD \| CONSTR \| METHOD | DETAIL: FIELD \| CONSTR \| METHOD |  |


---


## weka.gui.streams Class InstanceEvent

```
java.lang.Object
  java.util.EventObject
      weka.gui.streams.InstanceEvent
```

**All Implemented Interfaces:**: java.io.Serializable

---

``` public class InstanceEvent extends java.util.EventObject ```

An event encapsulating an instance stream event.

**Version:**
:   $Revision: 1.7 $

**Author:**
:   Len Trigg (trigg@cs.waikato.ac.nz)

**See Also:**: Serialized Form

---

| **Field Summary** | |
| --- | --- |
| `static int` | `BATCH_FINISHED`             Specifies that the batch of instances is finished |
| `static int` | `FORMAT_AVAILABLE`             Specifies that the instance format is available |
| `static int` | `INSTANCE_AVAILABLE`             Specifies that an instance is available |


**Constructor Summary**

`InstanceEvent(java.lang.Object source,
int ID)`
  
          Constructs an InstanceEvent with the specified source object and event
type


| **Method Summary** | |
| --- | --- |
| `int` | `getID()`             Get the event type |

**Methods inherited from class java.util.EventObject**

`getSource, toString`

**Methods inherited from class java.lang.Object**

`equals, getClass, hashCode, notify, notifyAll, wait, wait, wait`

**Field Detail**

### FORMAT\_AVAILABLE

```
public static final int FORMAT_AVAILABLE
```

:   Specifies that the instance format is available

    **See Also:**: Constant Field Values

---


### INSTANCE\_AVAILABLE

```
public static final int INSTANCE_AVAILABLE
```

:   Specifies that an instance is available

    **See Also:**: Constant Field Values

---


### BATCH\_FINISHED

```
public static final int BATCH_FINISHED
```

:   Specifies that the batch of instances is finished

    **See Also:**: Constant Field Values


**Constructor Detail**

### InstanceEvent

```
public InstanceEvent(java.lang.Object source,
                     int ID)
```

:   Constructs an InstanceEvent with the specified source object and event
    type

    **Parameters:**: `source` - the object generating the InstanceEvent: `ID` - the type of the InstanceEvent


**Method Detail**

### getID

```
public int getID()
```

:   Get the event type

    :   **Returns:**: the event type


---


|  |  |  |
| --- | --- | --- |
| **Overview** \| **Package** \| **Class** \| **Tree** \| **Deprecated** \| **Index** \| **Help** \| **Weka's home** | |  |
| **PREV CLASS**   **NEXT CLASS** | **FRAMES**    **NO FRAMES**     **All Classes** |  |
| SUMMARY: NESTED \| FIELD \| CONSTR \| METHOD | DETAIL: FIELD \| CONSTR \| METHOD |  |


---
